# Simulated Cranial Ultrasound for Longitudinal Learning (SCULL): A Simulated Task Trainer for Emergency Medicine Residents

**DOI:** 10.7759/cureus.114085

**Published:** 2026-08-06

**Authors:** Arrianna Mohammed, Richard Shin, Hannah Park, Kerianne Brady, Thomas Sanchez

**Affiliations:** 1 Emergency Medicine, NewYork-Presbyterian Queens, Flushing, USA

**Keywords:** pediatric skull fracture, pocus, simulation-based education, task trainer, ultrasound

## Abstract

Although point-of-care ultrasound (POCUS) is a useful imaging modality for evaluating suspected pediatric skull fractures, emergency medicine (EM) residents have limited opportunities to develop proficiency because these injuries are encountered infrequently during clinical training. To address this educational gap, we developed a low-cost, reproducible, and durable simulated pediatric skull model designed to replicate both intact and fractured cranial anatomy. The model was constructed from two inexpensive educational skull replicas and ballistic gel, with one skull manually fractured and reassembled using adhesive. Both skulls were coated with layers of ballistic gel to simulate scalp tissue. The total production cost was less than $200. The model was piloted in a small-group training session involving 14 EM residents. Participants received a didactic overview and then engaged in hands-on practice using ultrasound to assess both intact and fractured models. Survey data demonstrated strong educational value: 100% of learners found the session educational, 86% reported improved confidence in identifying cortical irregularities, and 93% expressed an intention to apply POCUS for skull fracture assessment in future practice.

## Introduction

Point-of-care ultrasound (POCUS) is a valuable diagnostic adjunct in the evaluation of pediatric head trauma. While CT remains the diagnostic gold standard, POCUS provides a safe, rapid, and noninvasive method for evaluating suspected skull fractures. Its specificity approaches 96% [1], but its sensitivity is variable, and successful use depends heavily on the skill and confidence of the operator [2]. Because pediatric skull fractures are relatively uncommon [3], emergency medicine (EM) residents have inconsistent opportunities for clinical exposure to these cases during training. This creates a gap in the acquisition of sonographic skills related to fracture detection. Head trauma is one of the most common reasons children present to the emergency department, accounting for a significant proportion of pediatric visits worldwide [4]. While most cases involve minor injuries, approximately 2-20% of children with head trauma are diagnosed with skull fractures [5], and between 15% and 30% of those with fractures have associated intracranial injuries [4]. The consequences of missed diagnoses can be significant, ranging from delayed recognition of intracranial hemorrhage to underestimation of traumatic brain injury severity.

Clinical decision rules such as Pediatric Emergency Care Applied Research Network (PECARN), Canadian Assessment of Tomography for Childhood Head Injury (CATCH), and Children’s Head Injury Algorithm for the Prediction of Important Clinical Events (CHALICE) have been validated to minimize unnecessary CT use [2]. While these tools reduce radiation exposure, their reliance on clinical signs and symptoms leaves room for diagnostic uncertainty. Furthermore, scalp hematomas may obscure physical findings, making fracture detection more challenging.

Several studies suggest high specificity and moderate sensitivity for detecting pediatric skull fractures, with specificity rates reported as high as 96%, a positive predictive value of 88%, and a negative predictive value of 97% [1]. However, effective use requires operator skill, familiarity with scanning techniques, and the ability to distinguish normal anatomy from fracture pathology.

Training EM residents in pediatric skull POCUS presents unique challenges. The incidence of fractures limits opportunities for direct patient-based learning. The annual incidence is estimated at 250 per 100,000, with approximately 10-30% of pediatric head injuries resulting in skull fractures. Simulation-based education offers a solution by allowing repeated, standardized practice in a safe environment.

Notably, several studies have demonstrated that EM residents and physicians can perform skull POCUS with high accuracy after focused training. One study showed that EM residents achieved 81.8% sensitivity and 100% specificity after training [6]. Another landmark study demonstrated that clinicians achieved 88% sensitivity and 97% specificity after just a one-hour focused ultrasound training session, with excellent interobserver agreement (κ = 0.86) [7]. This evidence directly supports the feasibility of teaching this skill to EM residents despite limited clinical exposure to actual cases.

To date, however, there has been a lack of widely available, cost-effective pediatric skull models for ultrasound training. The Simulated Cranial Ultrasound for Longitudinal Learning (SCULL) project was developed to fill this gap. We aimed to create an affordable, reproducible, and durable task trainer using accessible materials, enabling educators to expand exposure to this essential skill in EM training programs.

This project was previously presented as a poster at the American College of Emergency Physicians (ACEP) 2025 Scientific Assembly in Salt Lake City on September 9, 2025, and during the SimVentors Showcase at the International Meeting on Simulation in Healthcare (IMSH) 2026 on January 12, 2026.

## Technical report

Materials and tools

**Figure 1:**
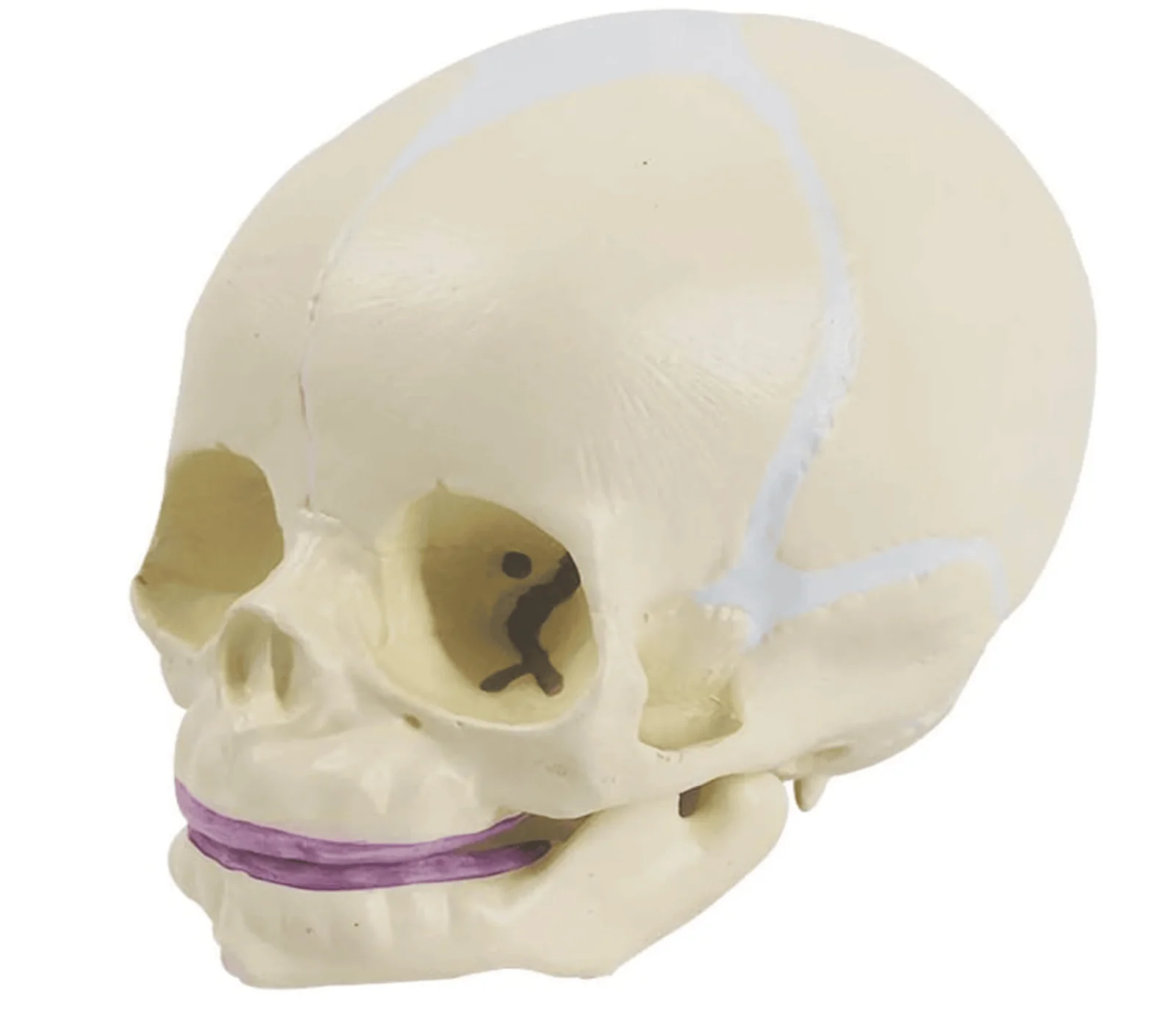
Educational pediatric skull replicas prior to fracture and gel coating.

For this project, two inexpensive educational pediatric skull models, each costing $29.99, were used as the foundation (Figure 1). These models provide anatomically accurate representations of cranial bones and sutures, making them appropriate for ultrasound-based training. Ballistic gel (10% Gel; Clear Ballistics LLC, Greenville, SC, USA) was selected to simulate scalp and soft tissue layers [6]. This commercially available gel is widely used in ballistic testing because of its tissue-like density and ultrasound compatibility [8-10].

Other required materials included adhesive glue to reconstruct fractured segments, a commercially available electric turkey oven for heating the ballistic gel, and basic laboratory supplies such as trays or molds to support skull positioning during the coating process. The overall cost of materials for producing two pediatric skull models was less than $200, making this model significantly more affordable to produce.

**Figure 2:**
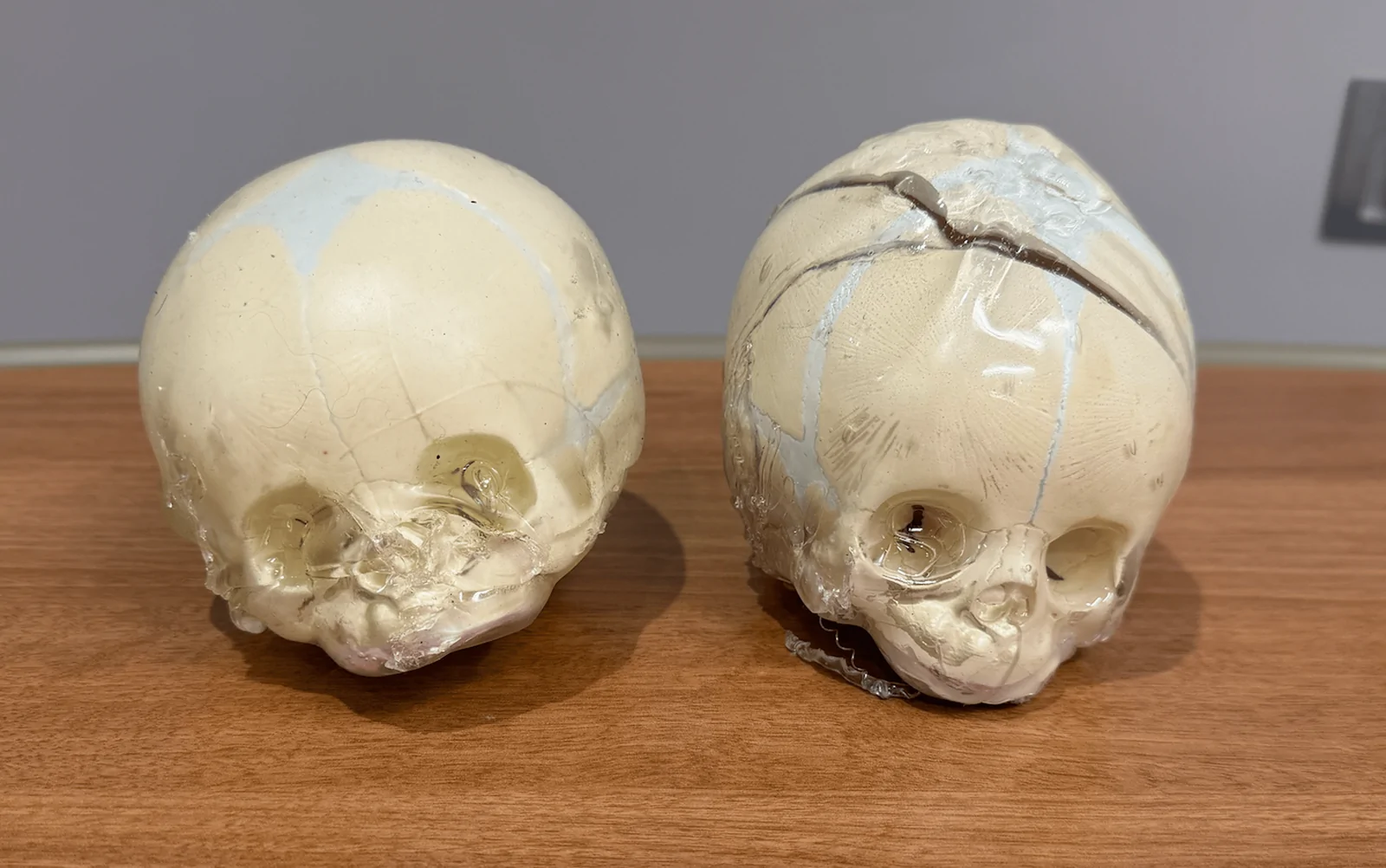
Educational pediatric skull replicas after the application of a multilayer ballistic gel coating. The left skull model demonstrates an intact skull without fractures, whereas the right skull model simulates a pediatric skull with a fracture.

The assembly process began with the preparation of the fractured skull. One of the pediatric skull replicas was manually fractured to create cortical irregularities and discontinuity in the bone structure. Fractures were induced using controlled blunt-force impact, a technique consistent with previously described methods for creating high-fidelity skull fracture ultrasound phantoms [11]. The fractured pieces were carefully reassembled using adhesive, leaving subtle but realistic fracture lines visible both externally and on ultrasound. The second skull remained intact, preserving normal anatomy for comparison. This dual-model approach allowed learners to appreciate the contrast between normal and pathological sonographic findings (Figure 2).

**Figure 3:**
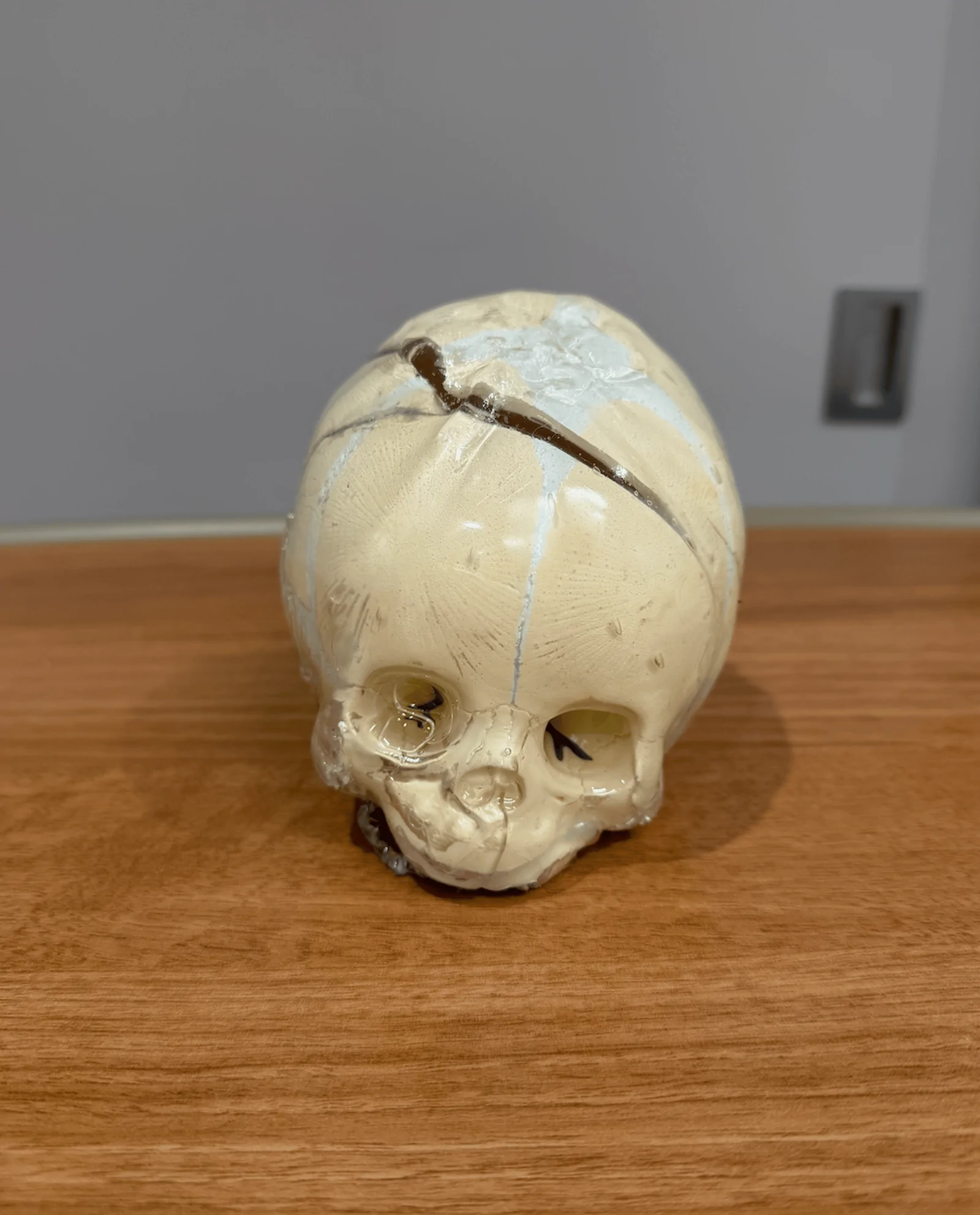
Educational pediatric skull replica after the application of a multilayer ballistic gel coating, simulating a pediatric skull fracture.

Ballistic gel was prepared according to the manufacturer’s instructions. Blocks of Clear Ballistics 10% Gel were placed into a commercially available electric turkey oven and heated to 250°F until fully liquefied. Each skull was dipped sequentially into the melted gel. For each coating, the vertex of the skull was immersed to the level of the orbital ridge before being inverted, ensuring even coverage across all surfaces. This process was repeated three times for each skull, producing three separate layers of ballistic gel. The layering was intentional to create a thicker simulated scalp that would both improve model durability and enhance the sonographic fidelity of soft tissue structures (Figure 3).

**Figure 4:**
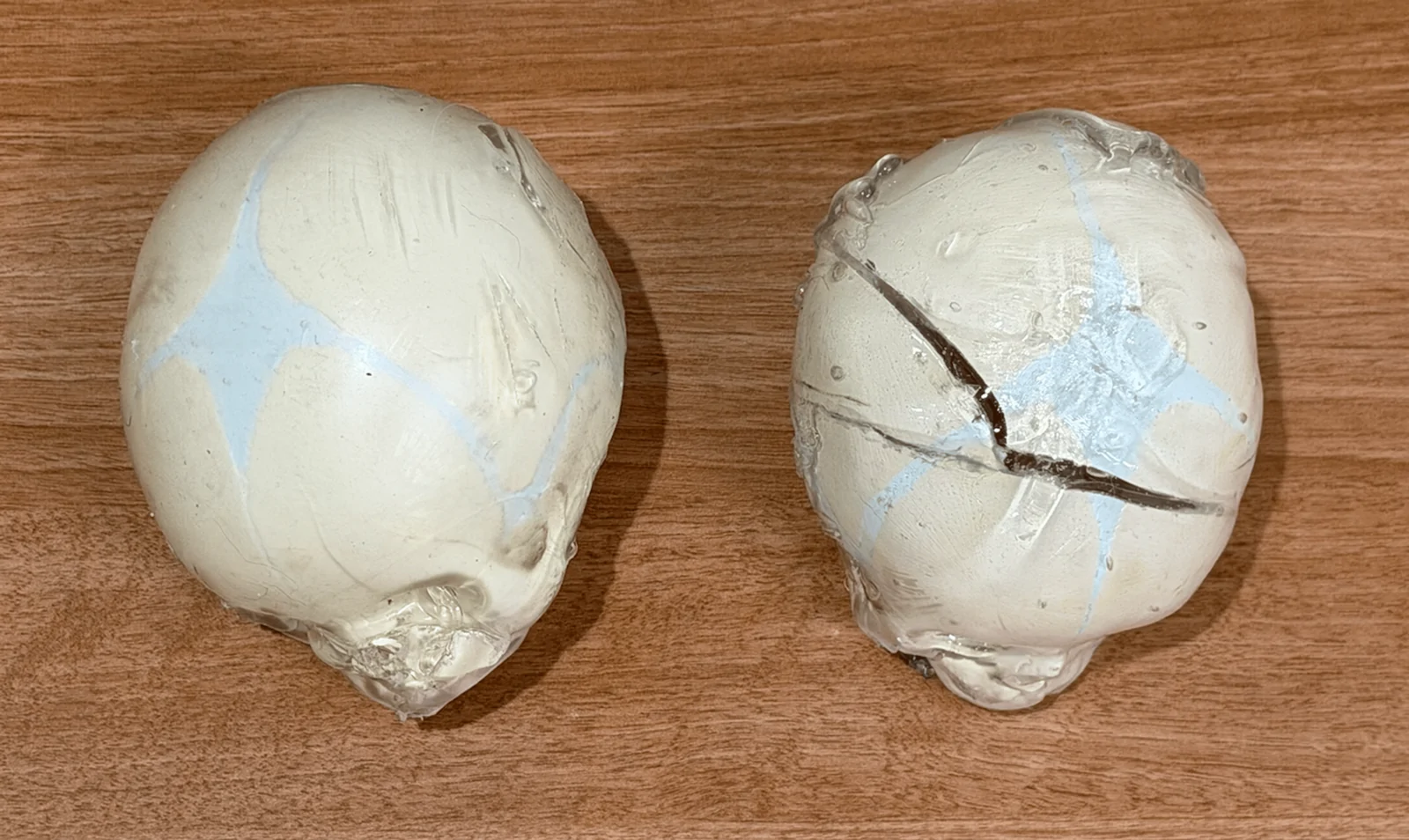
Educational pediatric skull replicas. Left: normal skull with ballistic gel coating. Right: fractured skull with ballistic gel coating.

Once cooled and solidified, the gel formed a firm but pliable outer layer that closely mimicked scalp tissue under ultrasound [8-10]. The final product consisted of two ultrasound-compatible models: one intact skull highlighting normal anatomy and pediatric suture lines, and one fractured skull demonstrating cortical irregularities and disrupted bone margins (Figure 4).

Educational utility and model functionality

The skull models were tested in a structured small-group session involving 14 EM residents from an Accreditation Council for Graduate Medical Education-accredited training program across all postgraduate training levels. The session began with a faculty-led didactic that reviewed the role of POCUS in pediatric skull fracture evaluation, discussed its strengths and limitations compared with CT, and highlighted the key sonographic features of intact bone versus cortical disruption.

Learners then rotated through a 30-minute hands-on workshop in which they used ultrasound to evaluate both the intact (Video 1) and fractured (Video 2) skull models. The gel layers provided realistic sonographic resistance, while the skulls allowed learners to identify anatomic landmarks, sutures, and simulated fracture lines. Faculty members provided direct supervision, feedback on probe technique, and guidance on image interpretation.

**Video 1:** Ultrasound examination of an intact pediatric skull model demonstrating a continuous cortical surface.

**Video 2:** Ultrasound examination of a fractured pediatric skull model demonstrating cortical irregularity and discontinuity.

Participants completed pre- and post-training surveys (Appendix A, Appendix B). Survey data collected immediately after the session revealed significant perceived educational value. All 14 participants (100%) reported that the session was educational. Twelve participants (86%) reported increased confidence in their ability to identify cortical irregularities using ultrasound. Only five residents (36%) reported prior experience with pediatric skull ultrasound, yet 13 (93%) indicated that they intended to incorporate this skill into their future clinical practice. The greatest gains in confidence were observed among junior residents with minimal prior ultrasound experience, highlighting the potential of this model to bridge educational gaps early in training.

## Discussion

The SCULL model represents an innovative, inexpensive, and reproducible approach to pediatric skull ultrasound training that can be used multiple times. Unlike high-fidelity commercial simulators, which can be prohibitively expensive for many residency programs, low-cost simulation models can be constructed for less than $200 and have demonstrated equivalent educational effectiveness [8-10]. Ballistic gel provides realistic sonographic properties, with acoustic characteristics that closely approximate those of human soft tissue [12,13]. The combination of intact and fractured skull models offers learners the opportunity to practice differentiating normal anatomy from pathology in a safe, controlled environment [14,15].

The evidence demonstrates that simulator fidelity does not necessarily correlate with learning outcomes. Multiple randomized controlled trials have shown that low-fidelity simulators are noninferior to high-fidelity simulators for teaching both technical and nontechnical skills, with studies reporting no significant advantage of high-fidelity over low-fidelity simulation (average differences of only 1-2%) [8-10].

The American College of Emergency Physicians guidelines recognize simulation as an important educational method for POCUS training, noting that it provides opportunities for deliberate practice and exposure to a wider spectrum of pathology than is typically encountered during clinical rotations [14,15].

Feedback from the pilot session demonstrated that even a single exposure to the model increased learner confidence in identifying skull fractures with ultrasound. A notable shift in self-reported confidence was observed between the pre- and post-survey assessments. When asked, “Are you able to identify cortical irregularities on skull POCUS?”, the majority of participants initially reported low confidence, with 57.1% selecting “not confident at all,” 28.6% selecting “not confident,” and 14.3% selecting “neutral.” Following the training intervention, no participants reported low confidence levels; instead, responses shifted to 14.3% selecting “neutral,” 57.1% selecting “confident,” and 28.6% selecting “very confident.” This finding is particularly significant given that the majority of participants had little or no prior experience with pediatric skull POCUS. By allowing repeated practice in a safe, controlled setting, the model enhances skill acquisition and promotes deliberate practice.

There are, however, limitations. The durability of the gel coating must be evaluated over repeated use, as ballistic gel can degrade over time. Additionally, while the model successfully simulates cortical irregularities, it does not replicate associated findings such as scalp hematomas, soft tissue swelling, or intracranial injury. Future iterations could incorporate variable gel thickness or embedded materials to simulate these additional features. Multi-institutional studies are needed to validate reproducibility and evaluate whether training with this model improves clinical performance.

Despite these limitations, the SCULL model fills an important gap in resident education. It provides learners with a reproducible opportunity to gain hands-on experience in a skill that is rarely encountered during routine clinical training. Furthermore, its affordability and accessibility make it an attractive option for integration into curricula at programs with limited resources.

## Conclusions

The SCULL model offers a novel, reproducible, and inexpensive solution for teaching pediatric skull POCUS. By combining educational skull replicas with ballistic gel layering, we created ultrasound-compatible models that replicate both intact and fractured anatomy. The model was well received by EM residents, improved learner confidence, and demonstrated feasibility for widespread implementation.

As simulation continues to play an increasingly central role in medical education, models such as SCULL represent an important step toward democratizing access to high-quality procedural training. With minimal cost, straightforward construction, and strong educational impact, this model provides educators with a practical tool to enhance resident training and improve preparedness in the diagnosis of pediatric skull fractures.

## Figures and Tables

**Figure 5:**
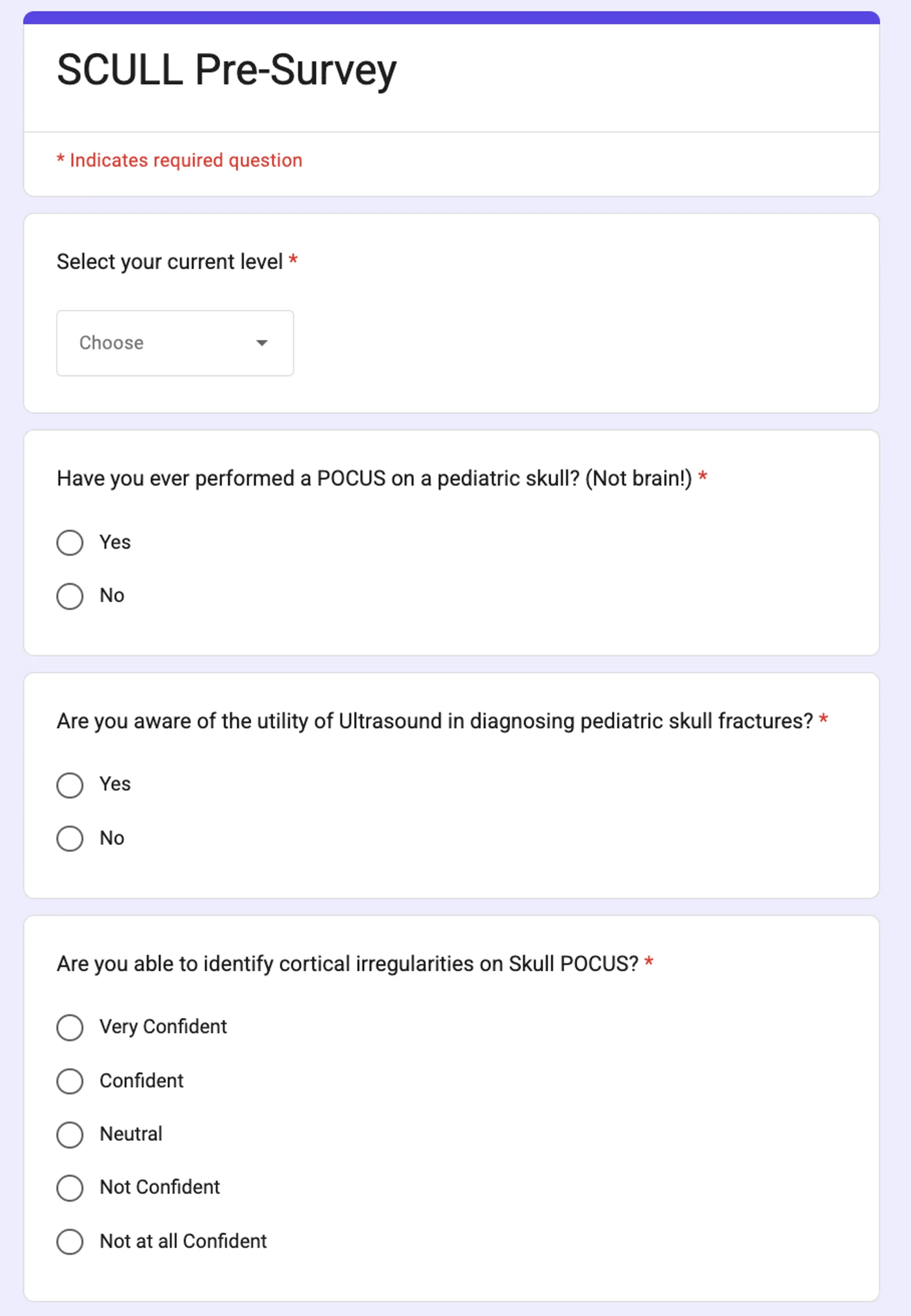
SCULL pre-survey

**Figure 6:**
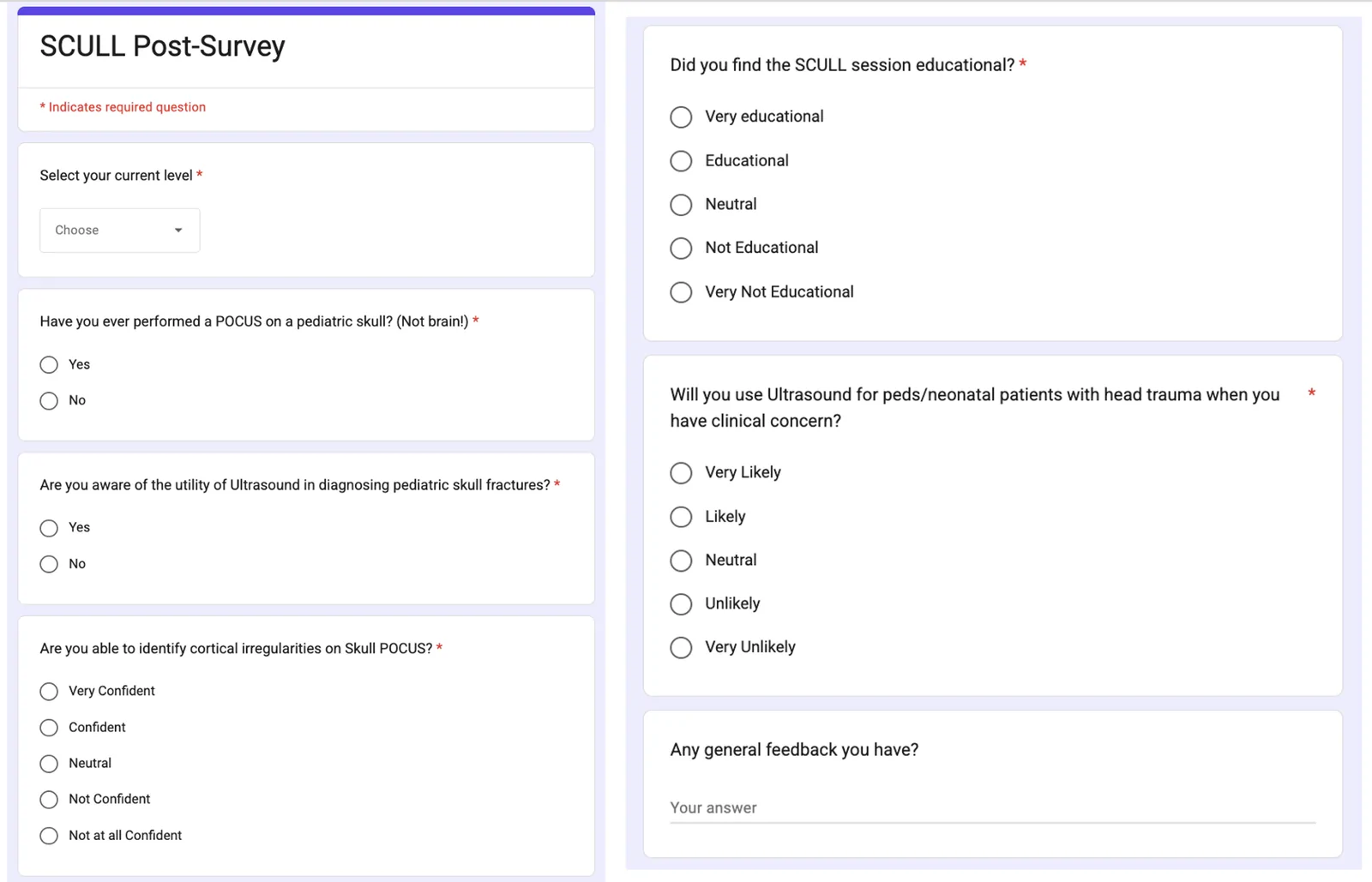
SCULL post-survey

## Appendices

Appendix A 

Appendix B
